# Recent Advances in the Treatment of Interstitial Lung Diseases

**DOI:** 10.7759/cureus.48016

**Published:** 2023-10-31

**Authors:** Aneesh A Bang, Sonali Bang, Arun Bang, Sourya Acharya, Samarth Shukla

**Affiliations:** 1 Medicine, Jawaharlal Nehru Medical College, Datta Meghe Institute of Higher Education and Research, Wardha, IND; 2 Ophthalmology, Sai Rugnalaya Hospital, Umarkhed, IND; 3 Orthopaedics, Sai Rugnalaya Hospital, Umarkhed, IND; 4 Pathology, Jawaharlal Nehru Medical College, Datta Meghe Institute of Higher Education and Research, Wardha, IND

**Keywords:** treprostinil, pirfenidone, nintedanib, lung transplant, interstitial lung diseases

## Abstract

Interstitial lung diseases (ILDs) are a group of disorders affecting the parenchymal tissue of the lungs. This disease leads to complications like pulmonary hypertension, heart failure, etc. that can affect patients. The etiological factors, clinical features, investigation methods, and diseases are conditions associated with ILD. The history of these conditions is of great value; any history of environmental and occupational exposure, medications, dust, or any toxic inhalation can be a predisposing factor. The CT scan is the investigation of choice in the case of ILD. This article states the recent advances made in treating interstitial lung diseases. The non-pharmacological and pharmacological management of ILD is discussed in the article. The discussion below concerns newer drugs approved by the FDA and their adverse effects, dosages, and contraindications. Below is a detailed conversation about ILD and the recent advances in treating this disease.

## Introduction and background

Interstitial lung disease (ILD) is a group of disorders that affect the parenchymal tissue of the lungs and alveoli. Lung parenchyma includes the alveoli, the alveolar epithelium, the capillary endothelium, and spaces between these structures [1]. ILD is also known as diffuse parenchymal lung disease. This condition involves a series of inflammations and fibrosis of the lung parenchyma, disrupting the normal physiology of the respiratory system. Thus, it is a restrictive type of lung disorder. There are various etiological factors for ILD. These can be classified into known causes and unknown causes. Known causes, such as environmental or occupational conditions, include allergens, coal dust, silica dust, and asbestos. Drug-induced due to drugs like nitrofurantoin, amiodarone, and chemotherapy and connective tissue disorders like rheumatoid arthritis, scleroderma, and Sjogren syndrome. Unknown causes or idiopathic causes don’t have a definitive cause.

The exchange of gases in the distal spaces is impaired as a result of structural remodeling. Previously, scientists and medical professionals believed that such remodeling was induced by a chronic inflammatory response; however, a more recent paradigm has been tissue injury that causes collagen-based fibrosis as a result of aberrant wound healing in these areas [2]. Various investigations can be performed, either to rule out the disease from other differentials or to determine the severity of the disease [3]. ILD has various systemic associations, and out of them, connective tissue disorders are seen to be most associated. The broad list of consequences that result from interstitial lung disorders can endanger patients' lives because they affect several different systems. The complication of pulmonary hypertension is one of these. Unlike pulmonary hypertension, which only affects the arteries supplying the lungs, systemic hypertension causes blood pressure to rise in all systemic arteries. The condition typically begins with tissue scarring or low oxygen levels, which constrict the blood vessels and reduce the amount of blood that can reach the lungs. The patient must first receive counseling before beginning non-pharmacological therapies. This improves the quality of life for the patients and slows the spread of the illness. It is recommended that the patient cut back on or give up smoking, learn about their allergies, and avoid exposure to them. Supplemental oxygen delivery can be used to increase exercise tolerance and minimize distress [4].

Lung transplantation extends and improves the quality of life for patients with ILD. Previously, there were three procedures, with each method having its advantages; these operations include single lung transplant, double lung transplant, and heart or lung transplantation. A single lung transplant was technically a more straightforward procedure and had less morbidity and mortality than a double lung transplant or heart and lung transplant [5]. The lung transplant can lead to primary graft dysfunction; this condition is often associated with prolonged and continuous ventilation of oxygen and the risk of infections by infective organisms. A single lung transplant is a very satisfactory procedure for patients with ILD. The recent drugs nintedanib, pirfenidone, and treprostinil have been approved by the government and have shown improvement in the conditions of patients. Though there are some side effects due to these drugs, they are more positive than negative [6].

## Review

Methodology

**Figure 1:**
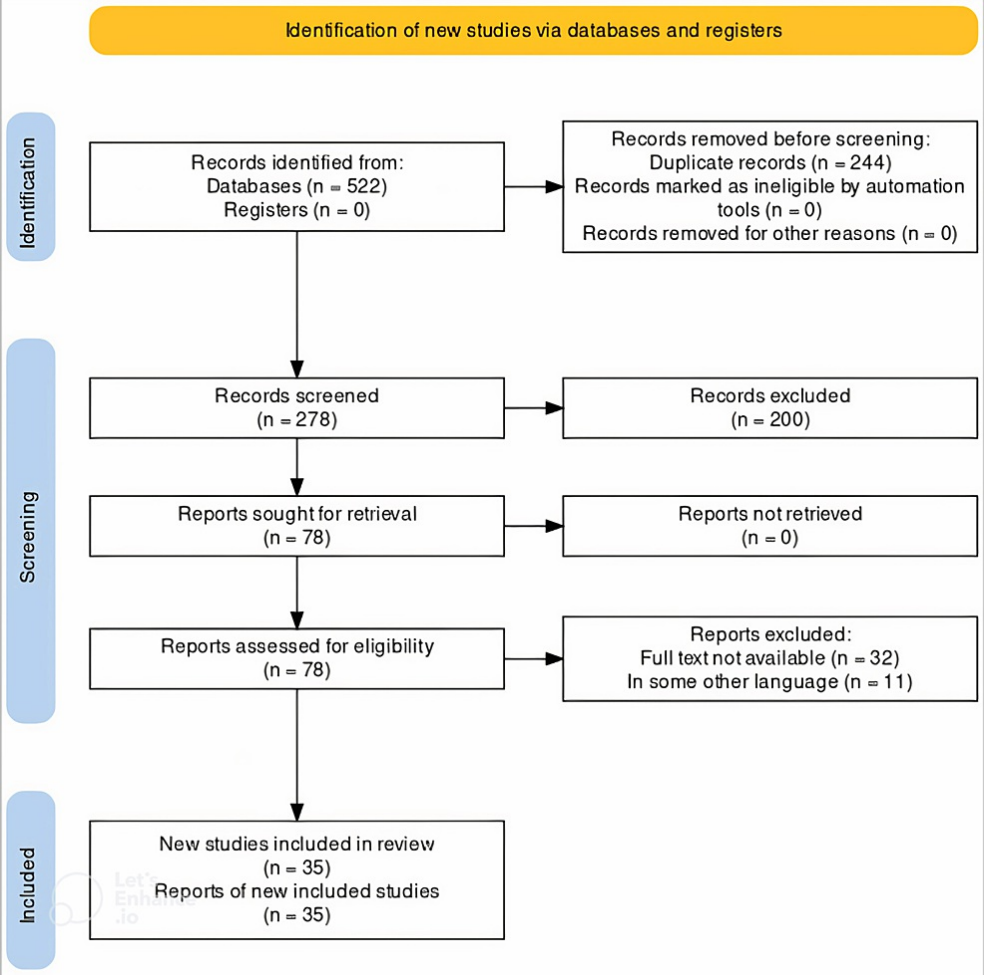
PRISMA flow diagram of recent advances in the treatment of ILD ILD: interstitial lung disease

The recent advances in the treatment of interstitial lung diseases and the new drugs were thoroughly reviewed using a literature search. A systematic search was undertaken through PubMed in June 2023 using keywords like “nintedanib", “interstitial lung disease” and “recent advances”. We also looked for key references to relevant studies in their respective bibliographies. Reference lists of pertinent publications and review papers were manually examined in addition to electronic database searches to find more studies. The selection process for the research that satisfied the inclusion criteria included observational studies, experimental studies, systematic reviews, and meta-analyses that looked at the relationship between drugs like nintedanib and their related outcomes. The inclusion of only peer-reviewed and published articles was taken into consideration. Two reviewers separately checked the titles, abstracts, and full texts of the retrieved papers to see if they met the inclusion criteria before including them, and any inconsistencies were settled by discussion and agreement. The steps for inclusion studies are depicted in Figure 1.

Pathology

There is an impairment of gaseous exchange in the distal spaces due to structural remodeling. Researchers and medical practitioners previously believed that a chronic inflammatory response caused such remodeling; however, a more contemporary paradigm has been tissue injury that results in collagen-based fibrosis that occurs due to abnormal wound healing in these spaces [2]. Complex cellular and molecular mechanisms (such as cell adhesion, migration, proliferation, apoptosis, extracellular matrix (ECM) biology, and phenotypic reprogramming) are involved in the healing of wounds and the development of fibrosis [3]. Adult ILDs have a higher prevalence of fibrosis than childhood ILD diseases. Numerous reviews about the pathogenesis of lung fibrosis (from an adult ILD perspective) are available. To understand the pathophysiologic abnormalities common to adults and children, starting with information about fibrosis is helpful.

Roles of alveolar epithelium and surfactant metabolism

The lung alveolar epithelium is the ultimate interaction between the bloodstream and the environment. The epithelium is crucial to maintaining good alveolar homeostasis because alveolar macrophages react to environmental insults by interacting with mesenchymal and vascular cells [4]. Whether intrinsic or extrinsic, stress on epithelial cells can affect the damage response, decrease innate immunity, cause epithelial apoptosis, promote aberrant epithelial-mesenchymal signaling, and cause a range of remodeling conditions, including fibrosis. We are learning a lot about how disorders of alveolar homeostasis lead to disease from the genetic abnormalities of surfactant proteins [3].

Clinical features

The most common symptom seen in patients is dyspnea of gradual onset, but sometimes they may also present with symptoms as simple as coughing [5]. Pleuritic chest pain is uncommon, but doctors can visualize such symptoms in conditions like sarcoidosis. Diffuse alveolar hemorrhage can lead to hemoptysis in patients with ILD [6]. The history of these conditions is of great value; any history of environmental and occupational exposure, medications, dust, or any toxic inhalation can be a predisposing factor. Genetics also play a role, so a family history of the disease is also essential. Symptoms of rheumatological disease can also be seen in ILD, as it is often associated with other systemic disorders.

Investigations

Blood tests are used to diagnose autoimmune illnesses and other inflammatory reactions to environmental immunogenic responses by detecting proteins, antibodies, and other indicators [7]. A computerized tomography (CT) scan is the first step in diagnosing ILD. It is the investigation of choice [8]. High-resolution computerized tomography can help determine the extent of pulmonary impairment caused by ILD and the treatment path for patients suffering from ILD. This has removed the need for invasive techniques for making the diagnosis. Echocardiogram findings may be normal. Lung function tests include spirometry and oximetry. Lung tissue analysis can help diagnose pulmonary fibrosis by examining the tissue taken by biopsy. The pathologist can take these tissue samples following bronchoscopy, bronchoalveolar lavage, or surgical intervention. A surgical lung biopsy is the current gold standard for obtaining tissue samples and diagnosing unclassifiable ILD. The first radiological investigation done in ILD patients is a chest X-ray, but it is rarely sufficient to make a diagnosis of the condition.

Systemic association of ILD

ILD associated with connective tissue disorders (inflammatory conditions) can affect blood vessels, tendons, bone, cartilage, and even some specific organs [9]. Some of these disorders include diseases like systemic lupus erythematosus, rheumatoid arthritis, systemic sclerosis, etc. In such cases, the lung manifests clinically as ILD, diffuse alveolar hemorrhage, aspiration pneumonia, etc. In these conditions, the major genetic factor has been identified as the activation of the MUC5B gene [10]. The alleles of this gene can relate to the destruction of lung function and the survival rate of patients. Clinical manifestations in such patients include constitutional and respiratory symptoms, which are primarily non-specific [11]. Constitutional symptoms like fever, fatigue, weight loss, and respiratory symptoms like dyspnea on exertion and dry cough are seen [9]. It can also involve various systems like the mucocutaneous, musculoskeletal, neurological, gastrointestinal, cardiac, and hematologic systems [12].

Complications

Interstitial lung diseases lead to a long series of complications that can be life-threatening to patients by involving multiple systems. Pulmonary hypertension is one of these complications. In the case of systemic hypertension, blood pressure rises in all the systemic arteries, but in the case of pulmonary hypertension, only the arteries that supply the lungs are affected. It usually starts with a tissue scar or decreased oxygen levels, which restricts the blood vessels and thus limits the blood flow to the lungs. Reduced blood flow, in turn, leads to a rise in the pressure of the pulmonary arteries, which can progressively worsen. Heart failure is also known as cor-pulmonary or right-sided heart failure. Heart failure occurs when the right ventricle gets less muscular than the left side of the heart. Eventually, the heart's right side falls, leading to pulmonary hypertension. Lung failure is the end stage of chronic interstitial lung disease. It occurs when there is a severe depression of blood oxygen levels and a rise in pressure in the pulmonary arteries and right ventricle, causing heart failure.

Recent advances in the treatment of ILD

Non-pharmacological Therapy

Patients initially need to be counseled and are started on non-pharmacological interventions. This helps the patients live a healthier life and helps to decrease the progression of the disease. The patient is advised to decrease or quit smoking; they are also advised to know about their allergens and prevent their exposure. Supplementary oxygen administration can be done to reduce dyspnea and improve exercise tolerance. Oxygen therapy helps patients with ILD improve their condition. It makes patients breathe and exercise easier, helps prevent complications due to low blood oxygen levels, and decreases blood pressure. It improves the patient's sleep and sense of well-being.

Surgical Management

Lung transplantation: This procedure extends and improves the quality of life for patients with ILD [13]. Previously, there were three procedures, with each method having its advantages; these operations include single lung transplant, double lung transplant, and heart or lung transplantation. A single lung transplant was technically a more straightforward procedure and had less morbidity and mortality than a double lung transplant or heart and lung transplant [14]. The lung transplant can lead to primary graft dysfunction; this condition is often associated with prolonged and continuous ventilation of oxygen and the risk of infections by infective organisms. A single lung transplant is a very satisfactory procedure for patients with ILD [15]. Recipients and donors are allocated for lung transplants by the lung allocation system. The lung allocation system gives a score from 0 to 100. Transplant candidates are scored depending on medical information such as forced vital capacity, pulmonary artery pressure, oxygen at rest, age, body mass index, six-minute walk distance, diabetes, functional status, and many more.

General contraindications for candidate selection should be respected in the donor shortage situation. The commonly faced contraindications in countries like India are reduced support from society, evidence of tuberculosis, deformities with the spine and chest wall, usage of abusive substances like tobacco or others, mental abnormalities with impaired ability to cooperate, untreatable significant organ damage, any previous history of malignancy, and obesity with a BMI >35kg/m2.

The patients are selected for the procedure of lung transplant if, during a six-minute walk test, the patient’s spO2 gets lower than 89%, if the patient is on long-term oxygen therapy, if the mean positive air pressure is more than 25 mm Hg, or if the diffusion capacity of the patient is less than 35% predicted.

Pharmacological treatment

*Nintedanib* 

It is a tyrosine kinase inhibitor used to manage ILD and its associated diseases by preventing the proliferation of fibroblasts.

Mechanism of action: It is a derivative of indolinone, which acts by binding to tyrosine kinase receptors [16]. This drug inhibits growth factors like fibroblast growth factor receptors (FGFR), vascular endothelial growth factor receptors (VEGFR), and Fms-like tyrosine kinase-3 (FLT-3), thus leading to restrictive activity over neoangiogenesis [16]. It prevents autophosphorylation and blocks the signaling cascades by binding competitively to the intracellular adenosine triphosphate (ATP) binding site of growth factor receptors [17]. In addition to blocking tyrosine kinase receptors, it also helps in blocking non-tyrosine kinase receptors, i.e., Src and Lck, directly, thus preventing fibroblast activation [18]. This ultimately results in inhibiting fibroblast proliferation and migration [19]. This decrease in lung parenchyma fibrosis helps advance toward managing cases like ILD and idiopathic pulmonary fibrosis (IPF).

Dosage: The drug nintedanib is presently available on the market in the form of soft capsules with 100mg and 150mg dosages. The recommended dose is 150 mg twice daily, to be taken whole with food. In case of any side effects, a reduction in the drug dose is advised to 100mg twice a day; in any case, an extra capsule is not recommended if any amount is missed. Instead, it is advised to continue the suggested schedule [20]. The drug is advised to be stored at room temperature and away from moisture; it is also recommended to keep it away from children and pets. If the drug has crossed its expiration date, the most efficient disposal method is a take-back program to the local pharmacy. When administering nintedanib, other precautions must be taken for drugs that are metabolized by CYP3A4 and P-glycoprotein enzymes, as this alters the bioavailability and metabolic activity of these drugs. Drugs like omeprazole, barbiturates, phenytoin, amoxicillin, azithromycin, ketoconazole, and rifampicin are some of the medicines that are given along with nintedanib that can affect its metabolism.

Adverse effects: Nintedanib's research was reported to involve various systems in the form of its side effects. The side effects seen due to this drug are nausea, vomiting, diarrhea, pain in the abdomen, and a decrease in appetite due to the involvement of the gastrointestinal system. An impairment of liver function along with an elevation of liver enzymes and a decrease in weight in the patient are also noticed. Coughs, respiratory tract infections, urinary tract infections, skin rashes, and ulcers are seen. Rarely, the central nervous system is also affected, but the side effects seen are mild in nature and present as mild headaches and fatigue. Patients identified with these side effects are advised to receive supportive treatment and are educated about the side effects of drugs. A dose reduction therapy is recommended, along with supportive management. These side effects are seen more commonly in females. The risk of hepatotoxicity is reduced when the dose is reduced to 100mg twice daily [21].

Contraindications: Nintedanib usage is not recommended in conditions of pregnancy and lactation. A highly effective contraceptive is advised for females who are in their reproductive age group, which should be started before medication and during pregnancy. The continuation of contraceptives must be done for at least three months after the last dose of nintedanib [22]. Breastfeeding during this nintedanib therapy is usually not advised by the practitioners. Moderate or severe liver impairment is also a contradiction in nintedanib treatment. Tobacco usage decreases the effectiveness of this therapy [23].

Pirfenidone

Pirfenidone is derived from pyridone and is synthetic. It is an antifibrotic and anti-inflammatory agent commonly used in cases of IPF.

Mechanism of action: Pirfenidone reduces the production of transforming growth factor-beta 1 (TGF-beta 1), which is a profibrotic and proinflammatory cytokine implicated in IPF. By inhibiting TGF-beta 1, it also inhibits the conversion of fibroblasts in the human lung into myofibroblasts, thus preventing collagen synthesis and extracellular matrix production [24].

Dosage: It comes in a capsule or tablet taken by mouth. It is usually taken with food three times a day, taken around the same time every day. 200mg is given as a recommended dose. It is given along with nintedanib as the first line of drugs.

Adverse effects: Pirfenidone has some common side effects, such as gastrointestinal disturbances, diarrhea, skin rash, nausea, and vomiting. Some less common side effects include black stools, loosening of the skin, chest pain, chills, a general feeling of tiredness or weakness, joint or muscle pain, etc.

Contraindications: Pirfenidone is contraindicated in patients with hepatic impairment [25]. Patients are advised to avoid sun exposure and ultraviolet (UV) exposure; patients with CrCl<80 are also in a contradictive condition during this therapy. Hypersensitivity reactions to the drug are also seen in patients. These drugs pose no risk of teratogenicity, and no problems are seen during pregnancy or breastfeeding.

Treprostinil

Treprostinil can be administered orally, by inhalation, or by infusion. It is a synthetic analog of prostacyclin, which causes vasodilation, that is, the treatment of ILD, or pulmonary artery hypertension.

Mechanism of action: It is a stable analog of prostacyclin, a prostaglandin derivative that acts as an anti-thrombotic agent and as a potent dilator of vessels [26]. These are used in conditions characterized by abnormally high blood pressure [27]. Treprostinil binds to the prostacyclin receptors, the prostaglandin D2 receptor 1, and the prostaglandin E2 receptor, activating them and elevating intracellular cyclic adenosine monophosphate (cAMP) levels [28]. This leads to constant activation and promotes the opening of calcium-activated potassium channels that lead to the hyperpolarization of cells. This facilitates the direct vasodilation of systemic and pulmonary arterial vascular beds, inhibiting the aggregation of platelets and inhibiting the inflammatory pathways.

Dosage: Treprostinil has various modes of delivery. Treprostinil can be administered orally, by inhalation, or by infusion. Continuous intravenous or subcutaneous infusions are two ways that medications might be given [29]. Treprostinil is present in Remodulin at concentrations of 1 mg/ml, 2.5 mg/ml, 5 mg/ml, and 10 mg/ml, delivered in 20-ml vials [30]. For new patients, the infusion rate is started at 1.25 ng/kg/min and can be decreased to 0.625 ng/kg/min if undesirable side effects are observed. The maximum weekly infusion rate for the first month is 1.25 ng/kg/min, and for the rest of the infusion, the maximum weekly rate of infusion is 2.5 ng/kg/min [29]. The inhalational form of this drug is accepted by the FDA and is sold under the name Tyvaso [31]. The drug in inhaled form is given by means of an inhalational device, and the patients are advised to use the drug four times a day with a break of four hours in between the doses. The FDA has approved the oral form of this drug, and taking it two to three times a day with food is advised [32].

Adverse effects: As this drug is a vasodilator, it can lead to an antihypertensive effect, which can affect the blood pressure in patients [33]. Due to the inhibiting effect on platelet aggregation, the risk of bleeding, and the patients taking anticoagulants [34], common adverse effects seen include pain, headache, cough, diarrhea, nausea, rashes, jaw pain, edema, and hypotension.

Contraindications: This drug has no contraindication when administered by subcutaneous, intravenous, or inhaled route [35]. A slight modification in doses is required for patients with hepatic dysfunction, but an oral form of this medication is contraindicated in patients with hepatic dysfunction. Side effects of this drug also include various systems, such as the gastrointestinal system, which presents with features like nausea, vomiting, diarrhea, pain in the abdomen, and a decrease in appetite [33].

The recent drugs along with their mechanism of action, dosages, side effects, and contraindications are summarized in Table 1.

**Table 1:** Summarizing recent drugs in the treatment of interstitial lung disease

Drugs	Mechanism of action	Mode of delivery	Dosage	Side effects	Contraindications
Nintedanib	Tyrosine kinase inhibitors	Soft capsules	150mg twice daily	Nausea, vomiting, impaired liver function, and urinary tract infections	Pregnancy and lactation
Pirfenidone	Anti-fibrotic and anti-inflammatory action	Capsules or tablets	200mg	Nausea, vomiting, skin rash	Patients with hepatic impairment
Treprostinil	Analogue of prostacyclin with anti-thrombotic properties	Infusion, inhalational and oral forms	1mg/ml, 2.5mg/ml, 5mg/ml	Nausea, vomiting, impaired liver function, skin rashes	No specific contraindications but oral forms are usually avoided in patients with hepatic impairment.

## Conclusions

ILD is a disease in which there is an impairment of gaseous exchange in the distal spaces due to structural remodeling. It is a chronic disease that can lead to fatal outcomes due to various complications in the long run. Advancements in research regarding the treatment of ILD are being conducted, and recently, some new FDA-approved drugs have been advised for the management of patients. Nintedanib is a tyrosine kinase inhibitor that can be given in their advised doses and is contraindicated in cases of pregnancy and lactation. Pirfenidone has anti-fibrotic and anti-thrombotic activity with no specific contraindications. Treprostinil is an analogue of prostacyclin that has anti-thrombotic properties and is usually avoided in patients with hepatic disorders. All of these new drugs have their own respective doses and specific modes of delivery, but some common side effects that they share include nausea, vomiting, infections, etc. These drugs have opened a new path for the treatment of ILD and have improved the recovery outcomes of patients.
